# Multidisciplinary meetings for lower urinary tract symptoms and benign prostate hyperplasia

**DOI:** 10.1002/bco2.70089

**Published:** 2025-11-03

**Authors:** Emilien Seizilles de Mazancourt, Nadia Abid, Gaele Pagnoux, Olivier Rouvière, Ricardo Codas Duarte, Alain Ruffion, Hakim Fassi Fehri

**Affiliations:** ^1^ Université Paris cité Paris France; ^2^ Department of Urology and transplant surgery Edouard Herriot Hospital Lyon France; ^3^ Department of urinary and vascular imaging Edouard Herriot Hospital Lyon France; ^4^ Department of Urology Centre Hospitalier Lyon Sud Lyon France

**Keywords:** Benign Prostatic Hyperplasia, Holmium laser enucleation of the prostate, Lower urinary tract symptoms, Multidisciplinary meetings, Prostatic artery embolization

## Abstract

**Objectives:**

To describe a single academic centre experience in the establishment of Multidisciplinary Meetings (MDM) for the management of male non‐neurogenic Lower Urinary Tract Symptoms (LUTS) and Benign Prostate Hyperplasia (BPH).

**Materials and methods:**

Retrospective analysis of all the cases discussed in MDM for LUTS/BPH in our academic centre over a year, and analysis of the implementation rates, reasons for failure of implementation and discrepancies between the proposal and the final decision.

**Results:**

Over one year, 108 cases were discussed. The implementation rate of the recommendation was 71% (77/108). The reasons for the failure of implementation of the recommendation were patient preference in 6 (5%), lost to follow‐up in 13 (12%), consultant decision in 4 (4%), patient deterioration or new comorbidities in 2 (2%), improvement of LUTS symptoms in 4 (4%) and the suggested technique was not available for technical reasons in 1 (1%). The consultant's proposal was validated in 98 cases (90.7%) and a different decision was made in 9 cases (8.3%).

**Conclusion:**

The establishment of a MDM for male LUTS/BPH is feasible and could contribute to the improvement of the management of patients. Further studies are needed to evaluate all its aspects.

**Abbreviations**
BPH: Benign Prostatic Hyperplasia
DRE: Digital Rectal Examination
EAU: European Association of Urology
ICIQ‐SF: International Consultation Incontinence Questionnaire Short Form
IIEF5: International Index of Erectile Function
HoLEP: Holmium Laser Enucleation of the Prostate
IPSS: International Prostate Score Symptom
LUTS: Lower Urinary Tract Symptoms
MDM: Multidisciplinary meetings
PAE: Prostatic Artery Embolization
US: Ultrasounds
USP: Urinary Symptom Profile

## 1 INTRODUCTION

Multidisciplinary meetings (MDM) are well established in oncology and are considered standard of care.^1^ They recently also became mandatory in France for the management of complex pelvic prolapse, and before the implementation of urethral slings due to issues surrounding the use of prosthetic material.^2^, ^3^ Putative benefits of MDM include improved communication between health professionals, improved clinical outcomes, educational opportunities for health care professionals, increased satisfaction and psychological well‐being of patients, increased recruitment into clinical trials and improved consistency, continuity and cost effectiveness of care.^4^

Every chronic disease can benefit from a multidisciplinary meeting when multiple specialties are involved in its management, such as urolithiasis,^5^ neurogenic lower urinary tract symptoms in patients with multiple sclerosis,^6^ and deep infiltrating endometriosis.^7^ We hypothesized that it would be particularly relevant for benign prostatic hyperplasia (BPH) due to the wide range of available medical and surgical treatment options,^8^, ^9^ the possible adverse events after surgical treatments,^10^ and because other specialists such as interventional radiologists can also participate in the management. Moreover, BPH can be associated with multiple diverse comorbidities such as urethral stricture, prostate cancer, haemostasis disorders, and it can appear in very elderly patients. In addition, the European Association of Urology (EAU) Guidelines recommends a mandatory multidisciplinary team approach between urologists and radiologists before performing prostatic artery embolization (PAE),^8^ and the Society of Interventional Radiology recommends a patient‐centred discussion,^11^ whereas the American Urological Association does not recommend performing PAE.^12^ Furthermore, novel minimally invasive treatments are constantly being developed; however, not all treatments have sufficient certainty of evidence and long‐term follow‐up to be specifically recommended in guidelines,^13^ and finally, guidelines are not patient‐centred decisions.

To our knowledge, no multidisciplinary meeting has ever been described for the management of BPH. The objective of this study is to describe our single academic centre experience in the establishment of MDM for the management of male non‐neurogenic Lower Urinary Tract Symptoms (LUTS) and BPH.

## 2 MATERIALS AND METHODS

We performed a retrospective analysis of all the cases discussed in MDM for LUTS/BPH in our academic centre. Ethical approval of this study was deemed not necessary by our ethical institution board because no data regarding patients was collected, and no intervention on patients was performed. The analysis was restricted to anonymized, aggregate‐level information about MDM discussions.

## 3 OUTCOMES

The primary outcome was the implementation rate of the decisions discussed in MDM. The secondary outcomes were the reasons explaining the failure of the implementation, the description of the cases discussed, the number of cases in which there was a discrepancy between the referring surgeon's proposal and the MDM's conclusion, the number of patients included in clinical trials and the frequency of patients undergoing surgery, mini‐invasive treatment or prostatic artery embolization that had been discussed in MDM.

## 4 MDM ESTABLISHMENT

One physician was responsible for the coordination and organization of the multidisciplinary meetings, with the aid of a secretary. The multidisciplinary meetings were held monthly. The physicians attending the MDM were senior urologists, radiologists and fellows and residents in urology and radiology. The meetings took place in person within the urology department, with the option to arrange videoconferences if necessary.

Patients discussed at the MDM were those with a potential surgical indication. Not all patients with LUTS/BPH were systematically presented. In addition, cases with complex diagnostic or therapeutic strategies could also be referred to at the discretion of the treating physician. The main objectives of the MDM were to validate the etiological assessment or suggest complementary investigations, to confirm or reconsider the indication for surgery and to review the surgical approach initially proposed or suggest an alternative treatment.

A computerized attendance sheet was established and integrated within the MDM report. The cases were prepared by the physician in charge of the patient prior to the discussion. To do so, a standardized computerized form was made available in the electronic medical record with a detail of all the information needed: medical history and in particular of prior urologic endoscopic treatment, anticoagulation and/or antiplatelet therapy, medical treatment of BPH, voiding mode, functional scores such as IPSS (International Prostate Score Symptom), ICIQ‐SF (International Consultation Incontinence Questionnaire Short Form), IIEF5 (International Index of Erectile Function), USP (Urinary Symptom Profile), desire to preserve ejaculations, findings of the digital rectal examination (DRE), results of the uroflowmetry and ultrasounds, prostate volume and measurement method, serum PSA, results of the cystoscopy, prostate MRI and prostate biopsies, whenever performed.

After the discussion, a written report was saved on the computerized patient file and a copy was sent to the patient's general practitioner by the secretary. The result of the MDM was also discussed between the patient and the treating physician during the following medical visit.

## 5 STATISTICS

The outcomes are presented with the frequency and percentage. The statistics are descriptive and were performed with R Studio (Posit Software, PBC).

## 6 RESULTS

Between the 1st January 2022 and 31st December 2022, 12 MDM were held, and 108 patients were discussed. An average of nine patients was discussed at each meeting. Ten patients were discussed for advice on the diagnosis, and 98 for advice on the therapeutic management.

The recommendations included various management strategies: holmium laser endoscopic enucleation of the prostate (61 cases), prostatic artery embolization (28 cases), minimally invasive treatment (Rezum® system, *Boston Scientific Corporation*, Marlborough, MA) (15 cases), medical therapy (3 cases), self‐catheterization (3 cases), sphincterotomy (1 case) and intradetrusor injection of botulinum toxin (1 case). Additionally, a therapeutic trial, such as the placement of a prostatic stent, was suggested in 7 cases (6%). Further diagnostic evaluations were recommended in 12 cases (11%).

The implementation rate of the recommendation was 71% (77/108) (Table 1). The reasons for the failure of implementation of the recommendation were patient preference in 6 (5%), lost to follow‐up in 13 (12%), consultant decision in 4 (4%), patient deterioration or new comorbidities in 2 (2%), improvement of LUTS symptoms in 4 (4%) and the suggested technique was not available for technical reasons in 1 (1%). Four patients were recommended for enrolment in clinical trials, and two patients were effectively enrolled in trials (50%).

**TABLE 1:** Results of multidisciplinary meetings.

Parameter	Number (n = 108)
MDM decision implemented	77 (71%)
Failure of implementation	31 (29%)
‐ Patient preference	‐ 6 (5%)
‐ Lost to follow‐up	‐ 13 (12%)
‐ Consultant decision	‐ 4 (4%)
‐ Patient deterioration or new comorbidities	‐ 2 (2%)
‐ Improvement in LUTS symptoms	‐ 4 (4%)
‐ Unavailable technique	‐ 1 (1%)
‐ Unknown	‐ 1 (1%)
Clinical trial enrolment/Clinical trial suggestion	2/4 (50%)
Advice for diagnosis	10 (9%)
Advice for therapeutic management	98 (91%)

The results are presented as numbers (percentage).

HoLEP: Holmium Laser Enucleation of the Prostate; MDM: Multidisciplinary Meeting; PAE: Prostatic Artery Embolization.

Out of 101 holmium laser endoscopic enucleation of the prostate performed in 2022, 46 (46%) had been discussed in MDM (Table 2). Sixteen cases (73%) out of 22 prostatic artery embolization performed in our centre had been discussed in MDM. All 15 patients who underwent mini‐invasive treatment had been discussed in MDM.

**TABLE 2:** Distribution of patients discussed in MDM among the patients treated.

	Number of patients discussed/number of patients treated
Patients undergoing HoLEP that had been discussed in MDM	46/101 (46%)
Patients undergoing PAE that had been discussed in MDM	16/22 (76%)
Patients undergoing miniinvasive treatments that had been discussed in MDM	15/15 (100%)

HoLEP: Holmium Laser Enucleation of the Prostate; MDM: Multidisciplinary Meeting; PAE: Prostatic Artery Embolization.

The concordance between the referring surgeon and the MDM's decision is reported in Table 3. Among the 108 patients discussed, the referring surgeon's proposal was validated in 98 cases (90.7%) and a different decision was made in 9 cases (8.3%). Additionally, among the cases where the MDM agreed with the surgeon's proposed management, alternative options were suggested for 17 patients.

**TABLE 3:** Concordance between the referring surgeon's proposal and the final decision of the MDM.

Decision outcome	Number (n = 108)
Proposal validated	98 (91%)
Alternative decision made	9 (8%)
Alternative option suggested (within confirmed proposals)	17 (16%)
Additional investigations requested	7 (6%)

MDM: Multidisciplinary Meeting.

## 7 DISCUSSION

In this study, we described the establishment and the results of MDM for BPH. To our knowledge, MDM meetings for non‐neurogenic male LUTS and BPH have never been described yet.

The implementation rate of the recommendation in our experience was 71%, largely influenced by the number of patients lost to follow‐up (13 patients, 12%). We cannot exclude the possibility that some of these patients sought care elsewhere, potentially due to disagreement with the MDM's decision. In comparison, the implementation rates of multidisciplinary meetings consensus in an Australian uro‐oncology setting were 91.1%^14^ and they reported that 67% of the uro‐oncological procedures had been discussed in MDM. The research on MDM is scarce, and there is not a good performance indicator of the value and efficacy of an MDM.^4^ We chose the implementation rates as the primary outcome because they are more often found in studies related to MDM. However, the recommendation issued by the MDM serves as guidance, rather than a mandatory directive. Moreover, we support patient decision, which can sometimes be in opposition to the recommendations. Patient Reported Outcomes Measurements (PROMs) before and after the establishment of MDMs could be a topic of future studies, as well as the comparison between patients that have been discussed in MDM with those who have not.

In our study, the consultant's proposal was validated in 98 patients (90.7%). These results are consistent with those of a British study on cancer MDMs, in which the consultant management plan aligned with the MDM consensus in 87.6% of cases. However, the management plan had been documented for only 15% of patients (81 out of 551).^15^

Van Belle showed that multidisciplinary meetings started to be organized in Belgium after they had been regulated by law and reimbursed.^16^ Indeed, MDM can be costly due to the high number of specialists required for the discussions,^15^ and it represents a supplementary workload. However, not all patients undergoing prostate surgery for bladder outlet obstruction require MDM discussion. It is most beneficial for complex cases, and it would be too time‐consuming to discuss all cases. Moreover, in our experience, the MDM meetings were held monthly, and as a result, some patients experienced acute urinary retention with unsuccessful bladder catheter removal, necessitating urgent surgery before being discussed at the meeting.

The standardized computerized sheet encourages consistency of care with all the relevant information required. Coercive measures could improve the inscription rate of patients.^17^ Further future improvements include financial incentives and dedicated time.^18^

The other physicians who could participate in a non‐neurogenic LUTS/BPH MDM include geriatrics specialists, specialists in physical medicine and rehabilitation, urodynamics specialists and palliative experts. Anaesthesiologists could also improve patients preoperative evaluation for high‐risk patients.^19^ Non‐medical participants such as nurses, social workers, psychologists and data managers could also be considered for participation in MDMs.^20^ The involvement of a pharmacist could also decrease drug‐related adverse events.^21^ Moreover, patients themselves could participate in their MDM to increase shared decision making.^22^ However, the number of participants should remain limited, as some, particularly physicians, might dominate the discussion.^20^, ^23^

Participants should bear in mind that participating in MDM implies medicolegal responsibilities.^24^ However, this should not deter physicians from participating but rather encourage a greater awareness and sense of responsibility, which both improve decision‐making. Moreover, the recommendations issued by the MDM are not necessarily meant to be strictly followed; they serve as guidance for management, with the final decision resting with the practitioner and the patient.

MDMs play an important role not only in optimizing patient management but also in medical education, particularly in academic settings. These discussions provide valuable opportunities for training by exposing residents and fellows to complex decision‐making processes, a benefit that warrants further evaluation. Additionally, such meetings can serve as an effective tool for identifying patients who may be eligible for clinical trials, ensuring that more patients have access to innovative therapeutic options. However, for a MDM to be truly effective, it must be supported by a healthcare facility with the necessary technical capabilities and expertise to offer a full range of therapeutic alternatives. This is the case in our institution, which is a tertiary centre with specialized teams and a comprehensive technical platform, ensuring that patients have access to all available minimally invasive techniques and expert‐driven decision‐making. In centres where these resources are lacking, referring patients to another centre with appropriate infrastructure should be considered to ensure optimal care.

This study has several limitations. First, it was a retrospective single‐centre audit, without a comparator group or before–after analysis. Consequently, no data on patients' outcomes, such as symptom improvement, quality of life or PROMs, were collected and no claim of clinical efficacy can be made. Second, we did not assess the satisfaction or perceived usefulness of the MDM for participants. As the majority of participants were the authors themselves, such an evaluation would have been subject to significant bias. Finally, because of its monocentric design, the generalizability of our results may be limited. Nevertheless, this pragmatic description provides initial insights into the feasibility and potential role of MDMs in the management of LUTS/BPH.

In conclusion, we have presented our experience in establishing a multidisciplinary meeting in the context of BPH/LUTS. While our results are encouraging and suggest that this approach benefits patients, further studies are needed to comprehensively evaluate all aspects of the MDM, including its impact on clinical outcomes, decision‐making processes and patient care.

## AUTHOR CONTRIBUTIONS

**ESDM:** Conceptualization; methodology; writing original draft. **NA:** Validation; writing review. **GP:** Methodology; writing review; data curation. **OR:** Supervision; writing review. **RCD:** Methodology; data curation. **AR:** Methodology; software; supervision; writing review. **HFF:** Conceptualization; writing original draft; data curation; supervision.

## CONFLICT OF INTEREST STATEMENT

The authors declare no conflicts of interest.
